# Pequi Pulp (*Caryocar brasiliense*) Oil-Loaded Emulsions as Cosmetic Products for Topical Use

**DOI:** 10.3390/polym17020226

**Published:** 2025-01-17

**Authors:** Tácio Fragoso Pereira, Huelinton Borchardt, Wvandson F. Wanderley, Ulrich Vasconcelos, Itamara F. Leite

**Affiliations:** 1 Programa de Pós-Graduação em Ciência e Engenharia de Materiais, Universidade Federal da Paraíba, João Pessoa 58051-900, PB, Brazil; taciofragoso29@gmail.com; 2 Graduação em Biotecnologia, Centro de Biotecnologia, Universidade Federal da Paraíba, João Pessoa 58051-900, PB, Brazil; hb@academico.ufpb.br; 3 Programa de Pós-Graduação em Ciência e Engenharia de Materiais, Universidade Federal de Campina Grande, Campina Grande 58429-900, PB, Brazil; wvandson.felipe@certbio.ufcg.edu.br; 4 Departamento de Biotecnologia, Centro de Biotecnologia, Universidade Federal da Paraíba, João Pessoa 58051-900, PB, Brazil; u.vasconcelos@cbiotec.ufpb.br; 5 Departamento de Engenharia de Materiais, Universidade Federal da Paraíba, João Pessoa 58051-900, PB, Brazil

**Keywords:** *Caryocar brasiliense*, cosmetics, sustainability

## Abstract

The pequi (*Caryocar brasiliense*) is a typical fruit from the Brazilian Cerrado. From it, pequi pulp oil is extracted, a valuable product for cosmetic applications due to its high levels of unsaturated fatty acids and carotenoids. Carotenoids are antioxidant compounds that are easily oxidized. To improve pulp stability, emulsification techniques with carboxymethylcellulose at 1% (*w*/*w*) were used to encapsulate the pequi pulp oil at 1, 3, 5% (*w*/*w*), and 8% (*w*/*w*) of polysorbate 80^®^ using a high-rotation mechanical stirrer. The pequi pulp oil was first characterized by FTIR and GC-MS. The results indicated the presence of chemical groups characteristic of the oil itself and the presence of a large proportion of fatty acids, which are essential for the maintenance of cutaneous hydration and the barrier, also acting in the tissue repair process. All emulsions presented stable over 120 days with slightly acidic pH values and were compatible with human skin. The droplet diameter was less than 330 nm, and the polydispersity index was around 0.3, indicating systems with low polydispersity. The Zeta potential (ζ) exhibited negative values sufficient to stabilize the emulsified systems. All emulsions behaved as non-Newtonian fluids, presenting pseudo-plastic and thixotropic properties that are considered important for topical applications.

## 1. Introduction

Consumer demand combined with new technologies in the development of products that are natural and sustainable has sparked discussion on the potential use of raw materials of plant origin, such as those found in the Brazilian Cerrado [1,2,3].

The Cerrado has great potential for the development of fruits and plants, with nutritional and therapeutic properties of relevance to the economy, culture, and health, and needs to be further explored. Among these fruits, the pequi (*Caryocar brasiliense*) stands out. From this fruit, pequi oil is extracted, which is rich in fatty acids such as palmitic, oleic, and linoleic acids, which have a number of biological and medicinal properties [4,5,6,7,8,9].

Pequi oil has been popularly indicated for the treatment of flu, respiratory and lung diseases, burns, gastric ulcers, and muscle and rheumatic pain [10,11]. Studies have shown its antifungal and antimicrobial activity, reducing effects on inflammatory processes, and its beneficial roles in tissue repair with faster closure of wounds, as well as its antioxidant activity. It is known that the presence of high levels of unsaturated fatty acids, such as oleic and palmitic, favors the healing process of wounds since they act as pro-inflammatory mediators, stimulate the production of growth factors, and promote fibroplasia and angiogenesis in the tissue [12,13,14,15,16,17,18]. In topical applications on intact skin, these substances are readily absorbed, forming a protective film, preventing abrasions, and providing local cellular nutrition [14], which is essential for maintaining skin integrity [15,16,17,18,19].

In addition, pequi oil also contains natural antioxidants, such as phenolic compounds and carotenoids. The color is due to the presence of carotenoids and is associated with the development and maintenance of human health, as well as with a reduction in the risk of degenerative diseases [20]. The actions and biological functions of these compounds are due to the system of conjugated double bonds in their structures, which makes them highly susceptible to degradation [21]. The main challenge in using these compounds, however, is their relatively low stability, which can cause changes in color and a loss of their biological activity. Among the methods used to reduce the instability of carotenoids are atomization, emulsion, extrusion, fluidized bed, coacervation, spray drying, molecular inclusion, and lyophilization, all of which can improve the stability of the compound, protecting against humidity, light, heat, and the oxidation of components [22,23,24,25,26,27]. Encapsulation has become an expanding area for reducing instability [20,21,28,29,30,31].

There are few studies reported in the literature regarding the encapsulation of pequi oil. These involve methods such as complex coacervation [32,33,34], production of microparticles by vibration nozzle [35], emulsion [36,37], emulsion followed by freeze-drying [38], emulsion followed by foam-mat drying [23], and the use of different materials for various applications. None of these make use of the materials treated in the present work, such as CMC and polysorbate 80^®^, nor do they use a high-rotation agitator for the preparation of emulsified formulations for topical application.

Pequi oil can be used in pharmaceutical formulations as emulsions [36,37], which are defined as thermodynamically unstable systems consisting of two immiscible liquids (oil and water), where the oil is dispersed in the aqueous phase (or vice versa) under the form of droplets with the help of surface-active agents. Emulsions have been one of the most promising alternatives for bioactive applications because they allow a modified release, both orally and topically, which can improve absorption and enhance the biological activity of a bioactive compound for the desired therapeutic purpose [39,40,41].

Sodium carboxymethyl cellulose (CMC) is an anionic polymer derived from cellulose and is very soluble in water. It is widely used in pharmaceutical applications as a thickener in emulsion preparations because it increases the viscosity of the continuous phase and promotes gel formation in an aqueous medium. In addition to stabilizing emulsions, increasing the viscosity of the aqueous phase and, consequently, delaying the movement of droplets and preventing their aggregation/coalescence, CMC enables the formation of stable systems through the steric effect, together with the polysorbate 80^®^ surfactant [42,43,44,45].

Many studies have reported the use of CMC in cosmetic and food formulations [46,47,48,49,50,51,52,53,54,55,56,57,58,59,60]. Reports based on emulsions containing pequi pulp oil and CMC, however, were not found in the literature. Therefore, the encapsulation of pequi pulp oil in carboxymethyl cellulose was chosen to be investigated as the focus of this work, where the chemical, physical, chemical, and rheological properties and morphology were studied.

This study of pequi pulp oil was aimed at its application for cosmetic/pharmaceutical purposes, signaling the use of natural, sustainable resources and, consequently, a contribution to regional socioeconomic development. In this context, there is great interest in introducing the use of this natural product in emulsified formulations containing carboxymethyl cellulose (CMC) so that potential derivatives with combined economic and therapeutic value can be produced.

## 2. Materials and Methods

### 2.1. Materials

Pequi pulp oil (PPO) (*Caryocar brasiliense*) was purchased from the São José market in Recife, in the state of Pernambuco, Brazil, and used without any processing. Polysorbate 80^®^, from Dinâmica Química^®^, was supplied by Cidalab (Campina Grande, PB, Brazil). Sodium carboxymethyl cellulose (CMC), CAS N° (9004-32-4), is an anionic polymer derived from cellulose with a substitution degree of 0.85 and is used as a thickener in emulsion preparations, provided by CRQ Eireli Chemicals Products (Diadema, SP, Brazil). All materials were used without any further purification, and the other reagents were of analytical grade.

### 2.2. Methods

#### 2.2.1. Sodium Carboxymethyl Cellulose (CMC) Solution

Aqueous solutions at 1% (*w*/*w*) of CMC were prepared under magnetic stirring at 60 °C, 1500 rpm, for 1 h until homogenization was complete. At the end of the process, the sample presented a gelatinous appearance. It was stored at room temperature in a glass tube and labeled CMC.

#### 2.2.2. Pequi Pulp Oil-Based Emulsions

The formulations were prepared by the (O/W) emulsification method, as reported in the literature, with some adaptations [61]. Emulsions containing 1, 3, and 5% (*w*/*w*) pequi pulp oil and 8% (*w*/*w*) polysorbate 80^®^ were selected for the study. First, oil was added, followed by the surfactant, and finally, distilled water, adjusting the final volume to 100% (*w*/*w*). This system was then subjected to mechanical stirring (MA 147, Marconi, São Paulo, Brazil) at 12,000 rpm for 10 min at ambient temperature. Finally, the emulsions were stored in glass tubes at ambient temperature and labeled PPOE1, PPOE3, and PPOE5 according to the oil concentrations 1, 3, and 5% (*w*/*w*), respectively.

#### 2.2.3. Pequi Pulp Oil-Based Emulsions Containing Sodium Carboxymethyl Cellulose

First, pequi pulp oil-based emulsions were prepared according to the method described previously (Item 2.2.2). The emulsions were subjected to 60 °C under magnetic stirring at 11 rpm. After reaching this temperature, 1% (*w*/*w*) powdered CMC was added, and the mixture remained in this condition for 1 h until complete homogenization. Afterward, emulsions containing CMC were labeled as (PPOE1 + CMC, PPOE3 + CMC, and PPOE5 + CMC) and stored in glass tubes at ambient temperature.

#### 2.2.4. Fourier Transform Infrared Spectroscopy (FTIR)

FTIR analysis was performed with a Bruker^®^ spectrophotometer, Tensor 27, using the attenuated total reflectance (ATR) mode (Billerica, MA, USA). The FTIR spectrum of pequi pulp oil was recorded at ambient temperature in the spectral range from 4000 to 600 cm^−1^ and a resolution of 4 cm^−1^.

#### 2.2.5. Gas Chromatography Coupled to Mass Spectrometry (GC-MS)

The pequi pulp oil was analyzed in a gas chromatograph coupled to the Shimadzu^®^ mass spectrometer (GC-MS, Tokyo, Japan), model GCMS QP 2010 ULTRA, equipped with a Perkin Elmer (Waltham, MA, USA) capillary column Elite 5 (5% diphenyl and 95% dimethylpolysiloxane) 30 m long with a 0.25 mm inner diameter and 0.25 µm film thickness. The carrier gas was helium at a constant flow of 1 mL·min^−1^, the injector temperatures were a split mode 1:20, and the interfaces were, respectively, 300 and 300 °C; the injection volume was 1 µL of oil diluted in hexane and dehumidified with sodium sulfate. The initial temperature was 80 °C and continued for 2 min until the final temperature of 300 °C for 5 min at a rate of 5 °C min^−1^. The masses detector was operated with an energy impact of 70 eV. Finally, the analysis result was obtained to identify the chemical composition of the fatty acids present in the oil in the study. All peaks are presented in the chromatogram. The identification of compounds present in pequi pulp oil was based on comparing the mass spectrum obtained for each compound with the spectra of the library present in the software of the equipment, the Nist08, and in the reference book by Adams (2017) [62].

#### 2.2.6. Macroscopic Evaluation

Pequi pulp oil-based emulsions (PPOE) and their respective CMC-containing emulsions (PPOE + CMC) were monitored for a period of 1, 7, 15, 30, 60, 90, and 120 days at ambient temperature in glass containers. Visual inspection confirmed the stability of the emulsions during the analysis period, considering the color, odor, phase separation, flocculation, polymer precipitation, coalescence, and creaming [63,64].

#### 2.2.7. pH Measurements

The pH of the emulsions was measured using a portable pH meter (Digital Meter, Pen Type Incoterm, Porto Alegre, RS, Brazil), previously calibrated with pHs 4 and 7 in buffer solutions. The measures were performed on the emulsions for a period of 120 days at ambient temperature and used in the stability study.

#### 2.2.8. Optical Microscopy (OM)

Analyses were performed using an optical microscope from Olympus BX41M-LED^®^ (Tokyo, Japan), operating with stream analysis software. A sample of 10 µL was deposited on a glass slide and covered with a coverslip to be analyzed at magnifications of 50× and 100×. Images were recorded with an Infinity 1 digital camera attached to the equipment. The emulsions were submitted for analysis on the 7th day.

#### 2.2.9. Rheological Test

To determine the viscosity and rheological behavior of the pequi pulp oil and emulsions, a rotational rheometer model Smart Swap AR 2000ex^®^ (TA Instruments, Alzenau, Germany) cone and 40 mm Peltier plates, operating with Rheology Advantage Data Analysis software, were used. The rheological test was performed according to the literature [65,66]. Measurements were determined using a sample drop at 25 °C, with a rotation speed progressively higher (1–50 rpm) to obtain the rising curve. The procedure was repeated in reverse with lower rotational speeds (50–1 rpm) to obtain the downward curve. A break of 1 min was used to measure the viscosity at 10 min intervals. The emulsions were examined after 24 h of preparation and stored at room temperature.

#### 2.2.10. Hydrodynamic Diameter, Polydispersity Index (PDI), and Zeta Potential (ζ)

The droplet hydrodynamic diameter and the polydispersity index (PDI) were determined by the dynamic light-scattering method at 25 °C and an angle of 90° using Brookhaven Instruments, Zeta PALS (Zeta Potential and Particle Size Analyzer, Holtsville, NY, USA). The instrument operates with a He-Ne laser and λ = 659 nm. Zeta potential measurements were carried out at 20.5 °C by the microelectrophoresis technique, submitting the sample to an electric field (11.02 V/cm), frequency (2.00 Hz), and voltage (4 V) in the Brookhaven instruments. Samples were diluted using 1:100 *v*/*v* (emulsion/distilled water) and analyzed in a quartz cuvette using distilled water as a reference.

#### 2.2.11. Scanning Electron Microscopy (SEM)

The microstructures of the pequi pulp oil-based emulsions and carboxymethyl cellulose were investigated by SEM. A sample drop was deposited on a glass slide attached to a copper sample holder and submitted to a drying oven at 40 ± 5 °C for 72 h. The samples were metalized by depositing a thin gold layer with a thickness of around 22.5 nm and a current of 25 mA for a time of 1 min and 30 s on a sputter coater (EMITECH, model K550X, Gongming, Shenzhen, China) and analyzed in an electronic scan microscope (FEI, model QUANTA 450, Hillsboro, OR, USA). The images were recorded through a secondary electron detector using an accelerating voltage of 10 kV, a current of 1.0 nA, and a working distance of 6.4 mm.

## 3. Results and Discussion

### 3.1. Fourier Transform Infrared Spectroscopy (FTIR)

**Figure 1:**
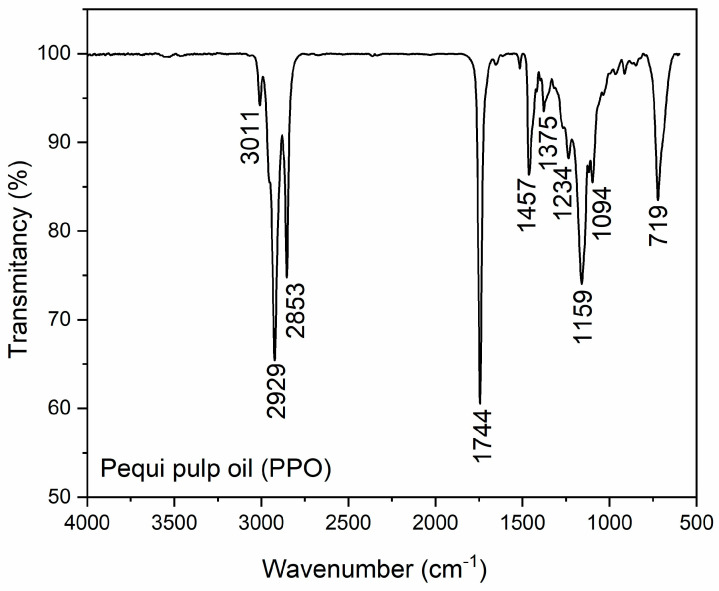
FTIR spectrum of pequi pulp oil (PPO).

In the pequi pulp oil spectrum (*C. brasiliense*) (Figure 1), an intense presence of lipid bands at 3011, 2929, and 2853 cm^−1^ can be observed, associated with the stretching vibrations of the C–H, –CH_2_, and –CH_3_ groups, respectively, which are characteristic of vegetable oils. These bands are also associated with the cis-alkene (–CH=CH-) present in unsaturated fatty acid molecules. The band at 1744 cm^−1^ refers to the stretching of the carbonyl group (C=O), corresponding to fatty acid esters [34,67]. Bands in the region of 1457–1159 cm^−1^ are assigned mainly to the deformations of the -CH_3_ and -CH_2_ groups, and the bands at 1234 and 1159 cm^−1^, respectively, correspond to the symmetrical stretching of the (C-CO-O) and (O-C-C) bonds referring to triglycerides and phytosterols [34,35,37]. At 719 cm^−1^, a bending vibration of the CH_2_ group is observed in an olefinic chain [35,67]. Similar results were reported by Sena et al. [68] when comparing the FTIR spectra of the oils extracted from the pulp of pequi.

It can be inferred that the presence of these groups provides the pequi pulp oil important properties that make it a potential nutritional source as well as a potential application in medicine, highlighting skin and wound treatments [35,69,70].

### 3.2. Gas Chromatography Coupled to a Mass Spectrometry (GC-MS)

The fatty acid composition of pequi pulp oil and other compounds was identified by GC-MS. From the photochemical review, it was possible to identify the relevant groups of metabolites related to different biological activities that constitute the active principles present in plants. This knowledge is fundamental to defining their application as a source of raw material for various segments, especially for the pharmaceutical industry [71]. The results of chemical composition are presented in Table 1.

Fatty acids represent the largest proportion of lipids found in pequi pulp oil. The main components were acids as follows: oleic (31.25%), linoleic (27.42%), palmitic (24.11%), and stearic (9.65%), as shown in Table 1. Oleic and linoleic acids are the major components in the composition of the oil [4,7,8,9]. A high presence of oleic monounsaturated fatty acid (C18:1) (omega 9) confers great benefits to human health, participates in the metabolism, and plays a key role in the production and synthesis of hormones. In addition, omega 9 has a cardioprotective effect that reduces triglycerides, LDL cholesterol, total cholesterol, and glycemic index, contributing to the prevention of heart disease [34,72].

In addition to oleic monounsaturated fatty acid being widely used as an additive in cosmetic emulsions, soaps, and detergents due to its softening properties, it is also used as a protector and regenerator for the damage caused by solar exposition and lubricity, recomposing the oiliness in dry skins, which enables its use as a raw material in cosmetics formulations and pharmaceutical products [16,17,18,19]. It also presents important activity in the induction of gene expression of B lymphocytes, which are involved in the defense and repair of tissues [73,74].

On the other hand, this oil also contains a high volume of saturated fatty acids, predominantly palmitic acid (Table 1). These concentrations are relatively high compared to other plant foods such as oilseed oils [75] and buriti pulp oil [76], other native fruits from the Cerrado. In short, monounsaturated and saturated fatty acids are well known to act as anti- and proatherogenic agents, respectively.

Linoleic acid (C18:2) (omega 6) is classified as a polyunsaturated fatty acid. It is a precursor of eicosapentaenoic acids (C20:3 n6) and docosahexaenoic acid (C22:6), which are associated with a decreased risk of cardiovascular and inflammatory diseases [77]. According to Kim et al. [78], linoleic acid favors the healing process and acts to protect lesions against chemical, enzymatic, and infections such as Staphylococcus aureus. The same result has also been observed by Declair [79], verifying growth inhibition of the same strain when using this fatty acid. Aside from these major constituents, malonic acid was also identified (3.74%), as well as geraniol (2.84%), γ-tocopherol (0.75%), and β-sitosterol (0.22%) in smaller proportions (Table 1).

Stearic acid (C18:0) is present in few species of plants and oils. Nowadays, it has considerable commercial importance. It is considered the most used acid in the cosmetic industry in the form of a thickener, emulsifier, and solubilizing agent in the production of creams, articles of rubber, resins, cosmetics, and textile products [80]. In the pharmaceutical industry, its main use is as a lubricant for capsules [81]. It is also applied in the form of derivatives in soaps and metallic salts [82].

Malonic acid is used in the pharmaceutical industry, mainly in the form of diethyl ester, in organic synthesis, with emphasis on the production of barbiturates and vitamins of complexes B1 and B2 [83].

In the pequi pulp oil composition, geraniol was identified to represent a volume of 2.84%. This constituent is present in citronella oil in addition to citronellol. These constituents act as attractants, repellents, and even as toxic substances to insects and microorganisms [84]. Those results corroborated the study carried out by Girão Filho et al. [85] that citronella powder is highly repellent to Zabrotes subfasciatus. Avelino et al. [86] studied the repellent action of several essential oils at a concentration of 0.05%. Among these oils, they observed that the fixed pequi oil (*Caryocar brasiliense*) results showed little attraction (28% of adult insects) but decreased the number of nymph production (65.22%) of Aphis craccivora (black aphid) in discs on lima bean leaves (*Phaseolus lunatus* L.).

Among the most important natural antioxidants are tocopherols, which are naturally present in most vegetable plants. This is a fat-soluble compound common in foods rich in unsaturated fats. There are four types as follows: α, β, γ, and δ, with α-tocopherol being the homologous antioxidant of the most efficient vitamin E in vivo [87]. Its antioxidant activity is mainly due to its ability to donate its phenolic radicals to free lipid radicals, inhibiting those present in body structures or nutrients exerting the same effect as vitamin E [88]. The present study found a content of 0.75% γ-tocopherol (Table 1), which has anti-inflammatory and antineoplastic properties [89].

The lowest proportion of antioxidants, 0.22%, found in the pequi pulp oil was β-sitosterol (Table 1). This was phytosterol, whose fatty substance is like cholesterol, naturally produced by plants and present in vegetable oils, seeds, grains, fruits, and vegetables. Plant cells synthesize a variety of sterol mixtures, particularly sitosterol and stigmasterol [90]. Phytosterols compete with dietary cholesterol and lower the intestinal absorption of bad cholesterol, LDL. Sterols may be present in free form and as fats esters and glycolipids [91], corroborating the result of the FTIR spectra discussed previously.

Pequi oil, rich in antioxidant and anti-inflammatory compounds, has been submitted to several studies, including those by master’s and doctorate students, in partnership between the company Naiak and the University of Brasilia. These studies demonstrated beneficial effects against body oxidation in athletes. In addition, they also studied the benefits related to its anti-inflammatory action, cardiovascular protection, prevention of atherosclerosis, and lowering blood pressure [92].

Identification of the constituents present in pequi pulp oil (*Caryocar brasiliense*) revealed the importance of this oleaginous typical fruit from the Brazilian Cerrado as containing essential compounds for health and presenting a broad spectrum of biological action.

### 3.3. Macroscopic Evaluation

**Figure 2:**
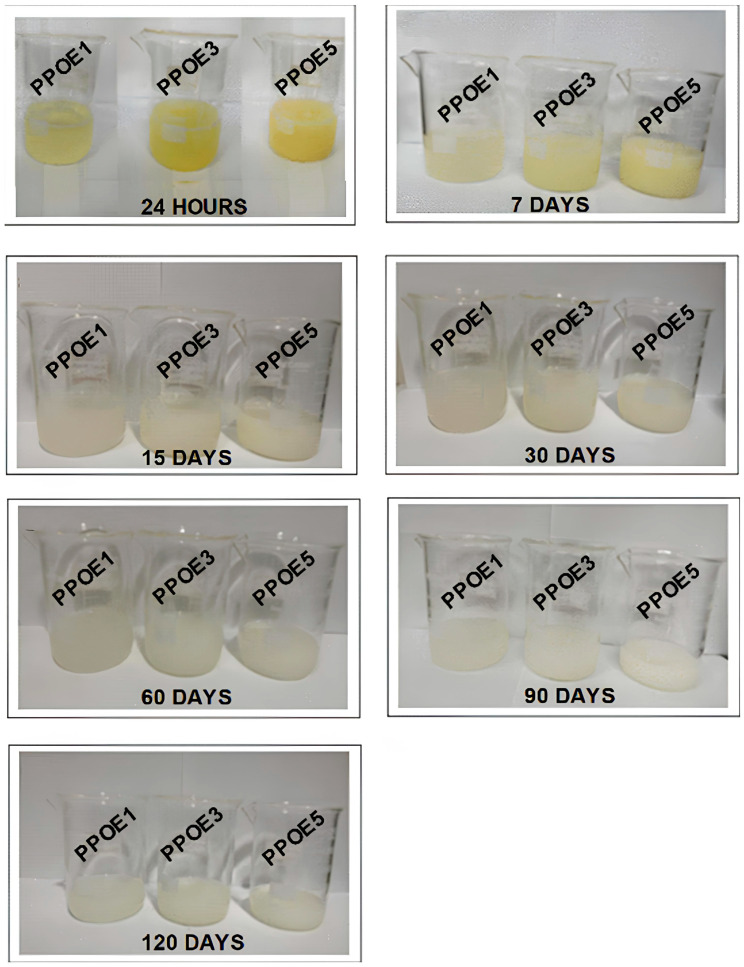
Macroscopic aspect of emulsions containing 1, 3, and 5% pequi pulp oil (PPOE).

**Figure 3:**
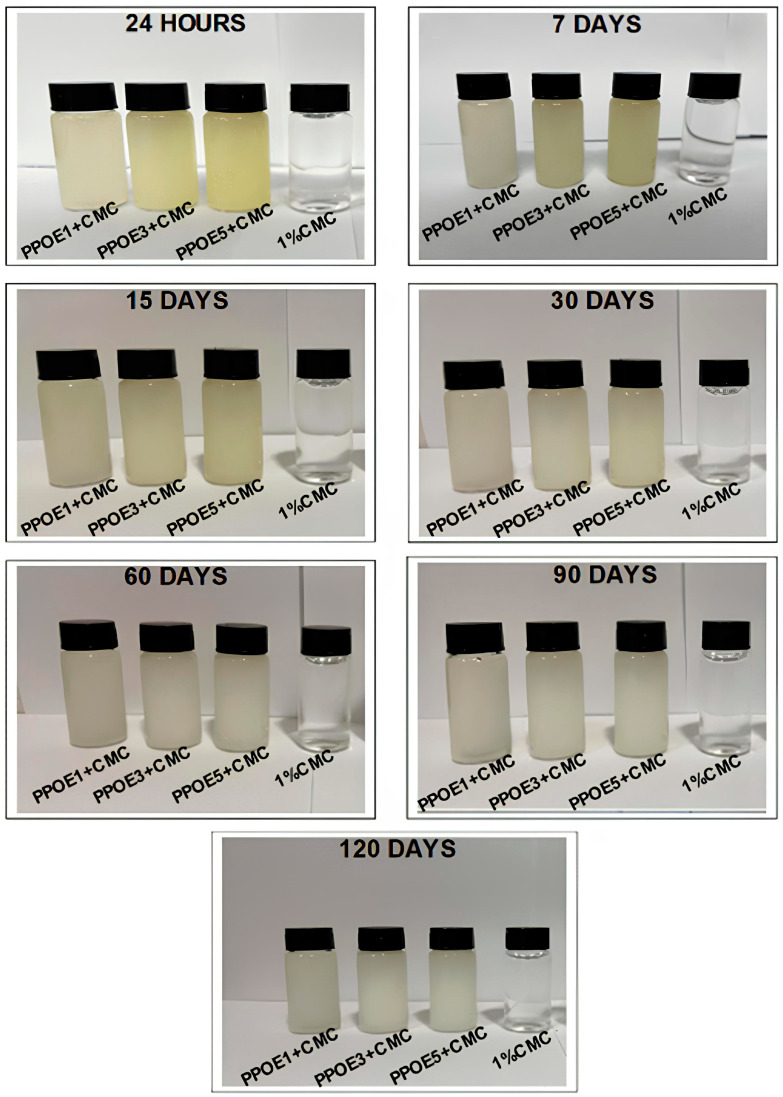
Macroscopic aspect of PPOE + CMC emulsions containing 1, 3, and 5% pequi pulp oil (PPOE) and pure 1% CMC solution.

Visual inspection over a period of 120 days was carried out to evaluate the stability of the emulsions formulated, considering the color, odor, phase separation, flocculation, polymer precipitation, coalescence, and creaming. The visual appearance of the oil/Tween 80/water (PPOE) emulsions with and without sodium carboxymethyl cellulose (PPOE + CMC) are shown in Figure 2 and Figure 3.

An analysis of Figure 2 shows that within 24 h, the emulsions containing 1, 3, and 5% (*w*/*w*) pequi pulp oil (PPOE) had become yellowish in color, becoming more intense as the concentration of oil was increased. This coloration is a unique characteristic of the oil extracted from the pequi pulp, as it contains carotenoids in its chemical composition. The presence of these gives the oil a color that varies from light yellow to dark orange, directly related to the content of the carotenoids present in the fruit [93]. These are the natural pigments in most Brazilian fruits, such as pequi, acting as antioxidants. The literature reports that the pequi *Caryocar brasiliense* species used in this study has a profile of total carotenoids superior to the *Caryocar villosum* [94], exhibiting antioxidant action that prevents lipid peroxidation and provides protection to the skin [66].

After 7 days, there was only a change in the color of the emulsions, yellow to gray, as of 60 days of analysis. After this time, the emulsions acquired a whitish color, regardless of the oil concentration. This whitish tone remained up to 120 days in the macroscopic analysis, showing no other change that could clearly show instability in systems such as phase separation, creaming, flocculation, and phase inversion. The loss of yellowish coloration was possibly due to a mild carotenoid oxidation in the formulations in this analysis. García et al. [95] stated that the oxidation of these components is accelerated by factors such as light, heat, and oxygen. According to Nascimento et al. [96], storage conditions influence the concentration of the carotenoids present in pequi pulp since light-protected packaging minimizes this loss.

On the other hand, all emulsions formulated in this study exhibited homogeneity and a characteristic pequi odor. Compositions with 1% oil were translucent, with a milky appearance (24 h; Figure 2). Emulsions containing 3 and 5% pequi pulp oil tended to be opaque (7 and 15 days; Figure 2), with or without a loss of color over time, indicating system stability at 120 days. This suggests that the choice of surfactant and concentration used (8% *w*/*w*), with mechanical agitation at high rotation (12,000 rpm), were able to achieve stable emulsions using different concentrations of the pequi pulp oil.

The addition of surfactants plays an important role in stabilizing the emulsified systems due to decreasing the interfacial tension of the system and forming an interfacial film with spherical and electrostatic properties around the internal phase droplets [97]. In this study, we used polysorbate 80^®^, a nonionic surfactant. The colloidal stability obtained for the different emulsions was due to the obtaining process, promoting steric stabilization with nonionic surfactants and/or polymers. 

Therefore, it was necessary to choose a surfactant suitable for the type of pharmaceutical form of intended use, bearing in mind that its use in microemulsion formulations for topical application is limited due to its toxicity, irritation potential, and action mechanism. In these cases, the use of nonionic surfactants is more frequent for emulsion synthesis [98].

When analyzing emulsions containing carboxymethyl cellulose (PPOE + CMC) (Figure 3), within 24 h, a slightly yellowish color was observed, proportional to the concentration of the oil in relation to the pure CMC solution, which exhibited optical transparency and was colorless. With the passage of time (7, 15, and 30 days), there was a total loss of the yellow color; the formulations started to present a gray color up to 120 days, as illustrated in Figure 3. This behavior was possibly due to carotenoid oxidation occurring in these formulations, as previously reported.

Incorporation of 1% CMC into the emulsions containing 1, 3, and 5% (*w*/*w*) pequi pulp oil was essential for achieving homogeneous, viscous formulations with a characteristic pequi odor, regardless of the presence of a yellowish tone in the emulsions during the whole analysis period. The CMC thickener, which is widely used in pharmacological applications, in addition to increasing the viscosity, promoted greater stabilization to the emulsified system, giving a positive effect to the final product. This product presents easy handling, good spreadability, and an ideal texture and appearance, promoting a beneficial effect in the treatment of skin. The development of emulsified formulations containing natural bioactive potentials, such as a pequi pulp oil, as used in this study, suggests a new pharmaceutical treatment with therapeutic action.

### 3.4. pH Measurements

pH is one of the simplest measurements but provides important results regarding the stability of emulsions. It can suggest the onset of an instability phenomenon, where a sharp decrease in this value indicates possible lipid oxidative degradation in the formation of the oil phase of the emulsion. This reaction takes place when atmospheric oxygen meets the sample, thereby producing peroxides and oxidative hydroperoxides. Lipid chain unsaturation can occur by free radicals, photooxidation, and enzymes such as lipoxygenase action [99].

The pH of the emulsified formulations was monitored at room temperature, with direct exposure to light. They were stored in glass containers for a period of 120 days, as illustrated in Table 2.

Emulsions (PPOE) containing 1, 3, and 5% (*w*/*w*) pequi pulp oil presented pH values at 24 h of 4.10, 4.35, and 4.43, respectively, proportional to the oil concentration. At 7 days, there was a slight increase to 4.50, 4.89, and 4.86, respectively. After this time, the pH values suffered a slight decrease, as observed in Table 2; by the end of 120 days of analysis, the emulsions showed the following pH values: 4.30, 4.52, and 4.52, respectively. The slight decrease after 7 days of analysis may be related to the loss of the yellowish color in the formulations associated with carotenoid oxidation, as discussed above in the section concerning macroscopic analysis.

Emulsions containing the CMC (PPOE + CMC) (Table 2) solution, when pure, had a pH of 7.1 (24 h). The incorporation of 1% CMC into emulsions containing 1, 3, and 5% (*w*/*w*) pequi pulp oil slightly increased the pH to 6.78, 6.89, and 6.85, respectively, within 24 h [100]. Therefore, the addition of CMC, which is an anionic polymer that is soluble in water [100] was chosen. This was unlike what was observed for the PPOE systems on the seventh day, where a slight increase in pH values was registered, followed by a slight reduction at 120 days of analysis. 

In the case of the PPOE + CMC emulsions, no such change was observed. From 24 h onwards, the pH values of the formulations decreased discreetly, reaching 6.58, 6.61, and 6.50, respectively, by 120 days. This behavior corroborates the reported results in the macroscopic analysis for these systems, where the CMC promoted a better stabilization of the formulations, showing a small variation (2.9–4.3%) in pH values during the analysis time. This value was less than that presented by the PPOE systems (9.7–12.4%) that are discussed above. From these data, it appears that the pH is strictly related to the excipients involved in the formulations and that the emulsions in this study did not present significant variations in the pH values over the 120-day period, being classified as stable, indicating that there was no degradation in the formulation’s components.

The skin’s surface pH is an important functional indicator of the health of the skin, whose value can vary from 4 to 6, depending on age and anatomical location. This particularity confers an important function barrier against bacteria and fungi proliferation, being essential for the process of tissue repair [101,102].

When there is a break in the continuity of the skin, resulting in a wound, its evolution depends on the pH value. For the healing process, the ideal situation is that the wound maintains the pH of the skin around it and that the environment is kept as unvarying as possible for the best healing process to occur. When a wound differs from the skin pH and assumes a value above the body pH, its healing is interrupted, and there is a greater risk of chronification and infection [103]. Of the several factors that change the healing process, one of them is the pH variation in wounds with an alkaline pH, indicative of infection, and is present in chronic wounds. A slightly acidic pH promotes a greater chance of faster healing.

The emulsions pH, in this study, ranged from 4.10 to 4.89 for PPOE (slightly acidic). The range was from 6.50 to 6.89 (acidic) for Percival et al. [104]. When adjusted to the pH closest to the skin, they are ideal for topical application and favorable for the cutaneous healing process of injured tissue, where the microenvironment of the wound plays a key role in the healing process.

According to Percival et al. [104], these different pH ranges are necessary for the various stages of healing and can determine wound healing. Therefore, the pH evaluation as a therapeutic target demonstrates that wound healing occurs most effectively in a slightly acidic environment, suggesting difficulty in healing chronic wounds that have a predominantly alkaline environment.

The evaluation of the pH in emulsion formulations is an important and necessary step in order to detect changes in the pH over time, ensuring that the pH is compatible with the components of the formulation and the location where application is desired [105].

The skin has a slightly acidic pH between 4.60–5.75, contributing to a protective barrier against bacteria and fungi when in contact with the skin’s surface [101]. All samples produced in this study had a pH compatible with the skin. The control and determination of the skin pH, especially for cosmetic or dermatological applications, are extremely important so that changes do not occur in the defense mechanisms [106,107,108].

According to Rebello [108], short-term products on the skin may be slightly alkaline (pH 8.0) since the alkalinity increases skin permeability. Products with pH values below 3.0 and above 8.0 should not be used on human skin, considering that they cause dryness of the skin due to keratin disruption or exacerbated removal of sebum.

### 3.5. Optical Microscopy (OM)

To evaluate the shape, size, distribution, and dispersion of droplets in the emulsions, the formulations were analyzed by optical microscopy at 50× and 100× magnifications.

**Figure 4:**
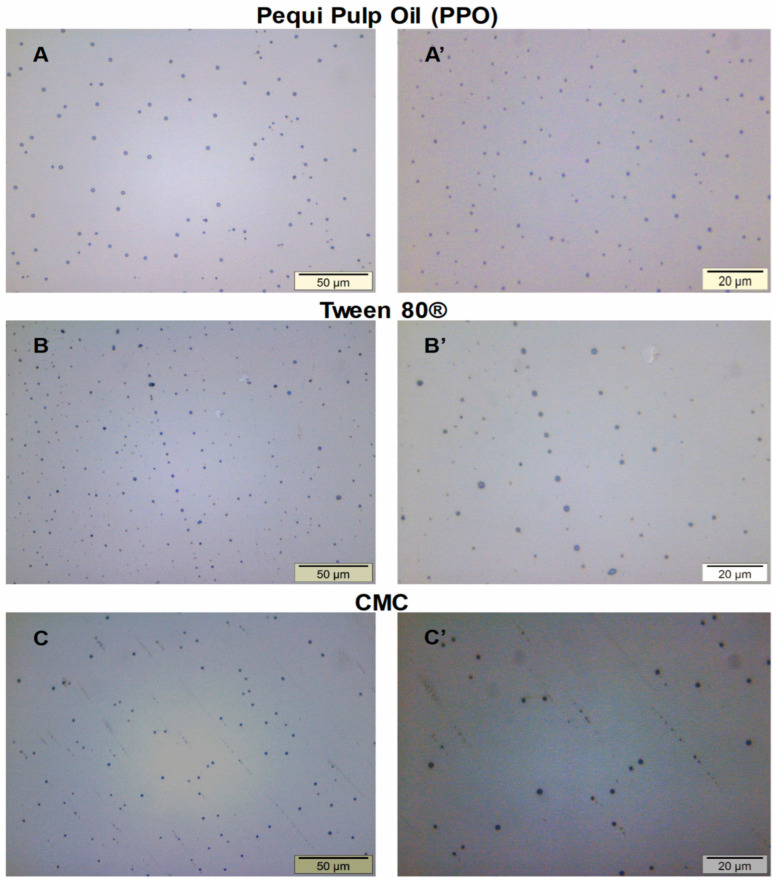
Micrographs of the (**A**,**A’**) pequi pulp oil (PPO), (**B**,**B’**) Tween 80^®^ surfactant, and (**C**,**C’**) CMC.

In the photomicrographs of pequi pulp oil (PPOE) (Figure 4), droplets are found in small, uniform sizes and are well distributed in the oily phase. These characteristics suggest safety regarding PPOE use in emulsified formulations for topical application, as well as for the prediction of system stability over time [109,110]. Similar behavior was also observed in the micrographs of the Tween 80^®^ surfactant used in this study (Figure 4).

It is important to emphasize here that the number of surfactants used in this study (8% *w*/*w*) was ideal for promoting possible kinetic and physical stability in the emulsions containing different concentrations of pequi pulp oil over a 120-day period of analysis. Additionally, the method and conditions of the process used proved to be efficient. This behavior corroborates the stability study of macroscopic analysis, already discussed before.

Emulsions with added polymers have a longer continuous phase, promoting viscosity, which reduces the fluidity and coalescence of the droplets, in addition to delaying the creaming. The increase in viscosity, the balance of droplet density, and dispersion prevent emulsion creaming to promote stability in the system [111]. On the other hand, an excessive increase in viscosity can lead to instability of the oil-phase droplets [112]. Sodium carboxymethylcellulose (CMC) was the polymer used in this study as a thickener, increasing the viscosity and contributing to the stability of the emulsified formulations [113].

**Figure 5:**
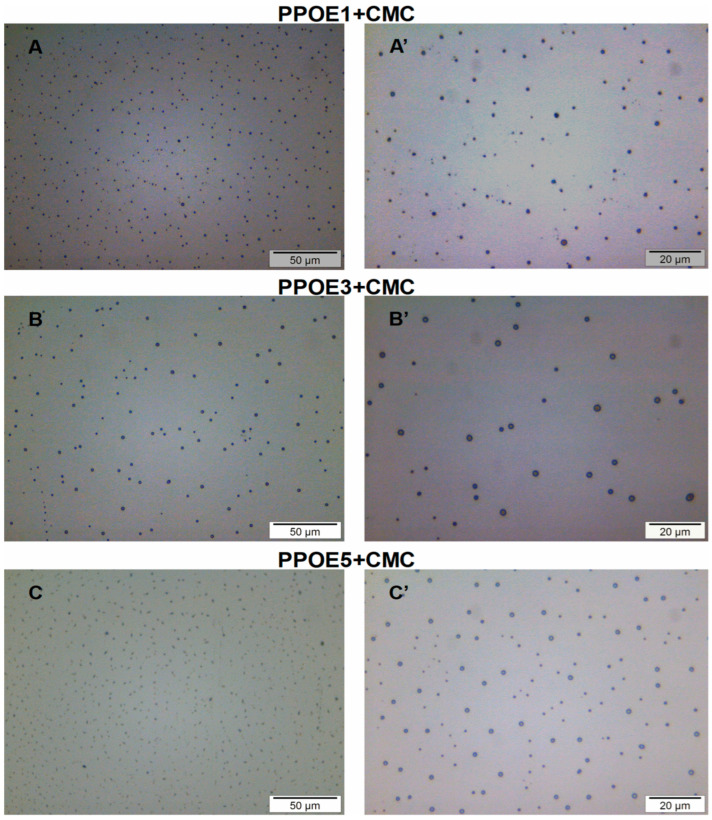
Micrographs of the PPOE + CMC emulsions containing (**A**,**A’**) 1, (**B**,**B’**) 3, and (**C**,**C’**) 5% pequi pulp oil.

The PPOE + CMC emulsions containing 1, 3, and 5% pequi pulp oil (Figure 5) displayed the presence of spherical droplets with different sizes and uniform dispersion, suggesting the formation of oil droplets in the CMC network. This morphology is better visualized for the emulsions PPOE3 + CMC and PPOE5 + CMC (100× magnification), whose droplet diameters were apparently larger than those of the PPOE1 + CMC sample. Similar morphology was reported by Marangon et al. [37] when studying emulsions of the chitosan/gelatin that contain pequi oil. The PPOE1 + CMC emulsion showed smaller droplet sizes; on the other hand, the PPOE3 + CMC sample exhibited a smaller number of droplets with relatively larger diameters when compared with those of samples containing 1 and 5% oil.

The droplet size is influenced by the qualitative and quantitative composition in the formulation’s preparation, as well as by the method of preparation. In general, emulsions with smaller variations in droplet sizes tend to be physically more stable; the amount of surfactant also influences this characteristic [114].

**Figure 6:**
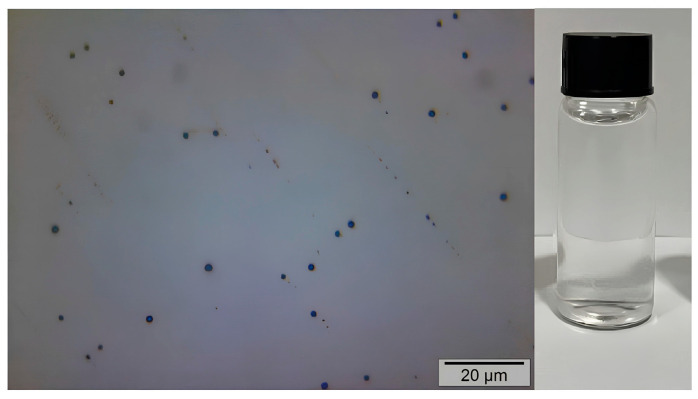
Optical microscopy image and visual appearance of the CMC solution.

Figure 6 presents the presence of CMC lumps, characteristic of total non-solubility of the polymer in the aqueous medium. These lumps have well-defined spherical formats that are small and evenly distributed in the system. Similar behavior was reported by [115].

The type of surfactant as well as the type of thickener used in the formulations, play fundamental roles in the stabilization of emulsified systems. The surfactant can be adsorbed on the surface or in the interface, changing its free energy [34,116]. In this study, Tween 80 was used, a nonionic surfactant whose inherent colloidal emulsion stability is due to steric stabilization. At the present time, CMC is widely used as a thickener in food additives and pharmacological applications to increase viscosity and improve the physical stability of emulsions, reducing oil/water interfacial tension and creating a physical barrier around oil droplets [45,46,49].

According to Tadros [110], smaller droplets are less susceptible to oscillation of the film formed by the surfactant around the internal phase droplets, consequently decreasing the occurrence of coalescence. The increase in the shear rate, however, increases the interfacial area without changing the surfactant used in this formulation. Given this, the higher shear rate may also contribute to a better distribution of the surfactant in the emulsion, consequently reducing the possibility of coalescence occurring. 

The surfactants and thickeners used in this study were crucial to the stabilization of the emulsions. Both these and the method used played a decisive role in the shear rate required for breakage and decrease in the size of the oil-phase droplets, along with the process parameters adopted in this study. This behavior can be proven with the stability study by macroscopic analysis as previously discussed, demonstrating physical stability for the different emulsified formulations.

### 3.6. Rheological Test

Rheology has been a topic of great importance for the pharmaceutical industry, bearing in mind that the type of surfactant, thickener, and concentration of vegetable oil used in emulsified formulations can directly influence the rheological behavior and physical stability of the finished product. Therefore, rheology study in formulations for topical use has become increasingly frequent by the scientific community since quality control, consumer acceptance, and effectiveness are fundamental, presenting a rheological adequate behavior of the application [101,117].

Among the various ways used to analyze trial data rheology, in this study, we opted for exploration, using stress curves of shear × shear rate (flow curves) and viscosity × shear rate (viscosity curves), through which it became possible to make a behavioral comparison dependent and independent of the time given for the emulsified formulations in this study.

**Figure 7:**
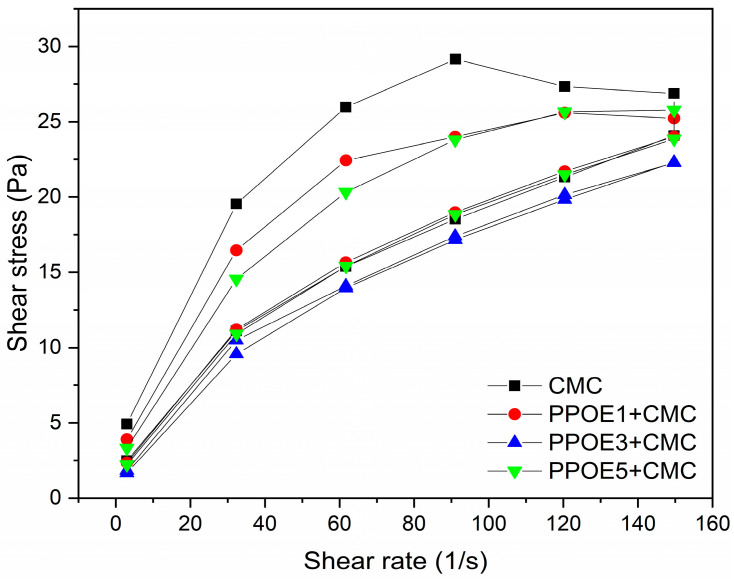
Flow curves as a function of shear rate at 25 °C for CMC solution and PPOE + CMC emulsions.

**Figure 8:**
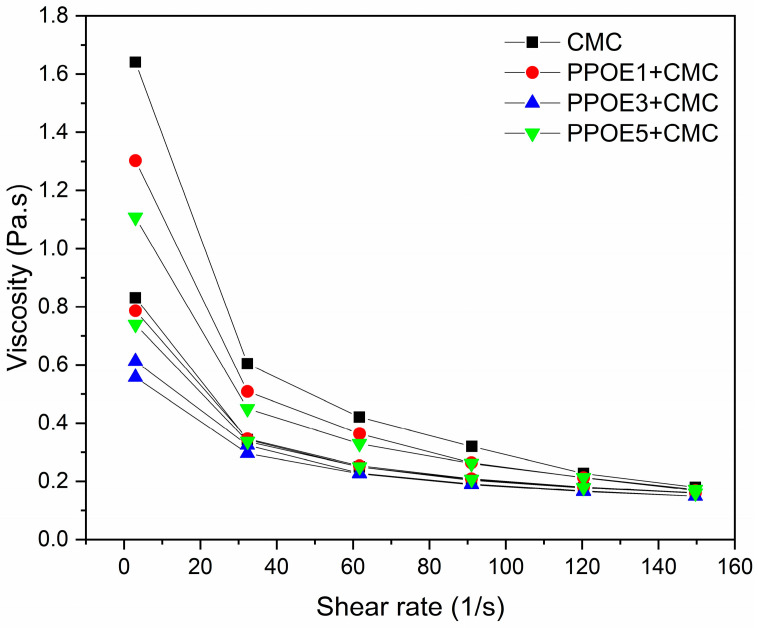
Viscosity curves as a function of shear rate at 25 °C for CMC solution and PPOE + CMC emulsions.

The flow curves of an emulsion indicate some of its most important physical characteristics in technical or aesthetic terms. Hence, there is a need to measure, adjust, and, if possible, predict such characteristics that are so useful and important for the formulation and application of the final product [118]. Shear stress causes deformation in solids and liquids; the solid deforms, and the liquid flows [119]. The flow curves and viscosity of the CMC solution and PPOE + CMC emulsions containing 1, 3, and 5% (*w*/*w*) pequi pulp oil are shown in Figure 7 and Figure 8.

All emulsified formulations (PPOE + CMC) containing 1, 3 and 5% (*w*/*w*) pequi pulp oil started at a given point, ascended and descended, and did not present a linear behavior (Figure 7) nor obeyed the law of Newton, being thus considered non-Newtonian fluids. A similar result was reported by Raiser et al. [119] when studying emulsions containing 5 and 10% pequi oil, which presented a non-Newtonian profile over the period of the study. 

Figure 8 shows that the viscosity for all formulations decreased with an increasing shear rate, characterizing it as pseudo-plastic fluid [66,120,121]. This type of behavior is very common in formulations when using gums and natural and synthetic polymers [122]. This behavior is attributed to the sodium carboxymethyl cellulose (CMC) used as a thickener in this work, which gave a gelatinous aspect to the formulations.

The curves in Figure 8 also show that the solution of CMC at 1% (*w*/*w*) has the highest viscosity, followed by PPOE + CMC with 1, 5 and 3% (*w*/*w*) pequi pulp oil (from top to bottom), respectively. It was expected that as the concentration of vegetable oil in the emulsified formulation increased, the viscosity would decrease. The opposite result was reported by Marangon et al. [37] when studying emulsions of chitosan/gelatin containing 1, 2, and 3% pequi oil. In this study, the addition of pequi oil led to an increase in the viscosity of the emulsions as the concentration tripled from 1 to 3% oil. This increase is a direct indication of a higher interfacial viscosity when there are higher oil concentrations, reflecting stronger interactions between the molecules present in oil and emulsions [123]. Increases in the viscosity of Pickering emulsions with an increasing oil-phase content were also reported by Li et al. [124]. These results make it clear that the viscosity of the emulsions will suffer variations due to the components present in the formulations, as well as possible interactions between them in addition to the oil concentration.

In addition to pseudo-plasticity as defined above, most formulations showed thixotropic behavior. Thixotropy is a variable time-dependent viscosity. This parameter provides information about the capacity and the time required for the emulsion to return to its structure after the removal of tension. The degree of thixotropy is assessed by the resulting area between the ascending and descending curve, as shown in the rheogram of Figure 7, called hysteresis [66,121].

Figure 7 also shows that the concentration of pequi in the pulp oil formulation greatly influenced the thixotropic characteristic of the final product: formulations with 1 and 5% oil were the ones that presented greater thixotropy compared to those with 3% oil. This behavior can be evidenced by a qualitative analysis of the hysteresis areas of the flow curves for all the formulations (Figure 7), where there are large areas for the solution of Pure CMC, followed by compositions containing 1 and 5% oil. The sample with 3% oil presented the smallest hysteresis area of all, barely perceptible. This was not considered thixotropy due to the variation between the ascending and descending curves not exceeding 10% of the equipment error limit. Similar behavior was observed by Pianovski et al. [66] in their studies using 10% (*w*/*w*) pequi oil in cosmetic emulsions.

In dermo-cosmetics as well as in pharmaceutical formulations for topical use, products with thixotropic behavior are desirable because they are more fluid, easy to spread, and the original viscosity is recovered as soon as the application has terminated, preventing the product from spreading over the skin [101,125]. On the other hand, formulations that have a low thixotropic value do not spread easily on the skin, promoting slow recovery. Further, very low values will cause difficulty for an even spread over the skin [125]. Emulsified formulations containing 1 and 5% pulp oil of pequi showed thixotropic behavior in different degrees, demonstrating a wide range of applications.

Another very important factor when choosing thixotropic formulations is the “shelf-life”, which tends to be longer due to the viscosity being constant over time, without promoting phase separation, leading to the stability of the formulation [117].

The rheological studies also allow inferences about the formulation’s stability according to the fluid type. The literature reports that obtaining microemulsions with a Newtonian profile and linear viscosity presented long-term stability [126,127,128]. On the other hand, Jiao and Burgess [129] analyzed the stability and the rheological behavior of emulsions containing different surfactants, Span 83 and Tween 80, which exhibited non-Newtonian behavior and greater viscosity. The addition of co-surfactants and/or oil phases can lead to a reduction in viscosity [116,130]. Acceptable stability can be observed in the study’s formulations, even showing non-Newtonian behavior for the analyzed time.

### 3.7. Zeta Potential (ζ), Polydispersity Index (PDI), and Droplet Hydrodynamic Diameters

Zeta potential (ζ) data, hydrodynamic diameter, and the polydispersity index of the particles present in the emulsions formulated and analyzed at 49 days are shown in Table 3.

**Figure 9:**
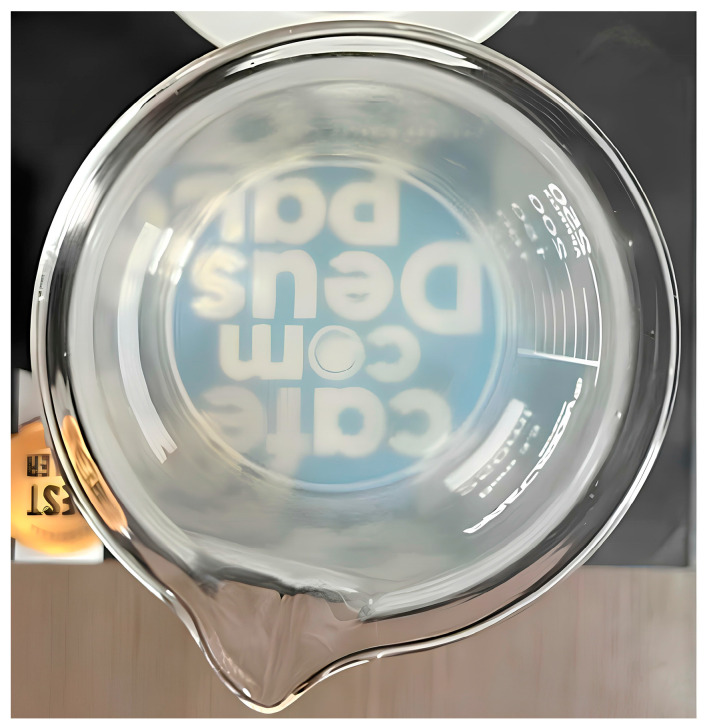
Photo of the top view of the PPOE1 emulsion, demonstrating the visible light translucency of the system.

One of the most influential parameters of the ζ is the pH. The pH provides information about the positive or negative charges that will interact with emulsion particles [131,132,133]. Pequi pulp oil, in this study, presented a pH of 5.2. By mixing it with the surfactant (polysorbate 80^®^) and aqueous phase for preparing the emulsified formulations, the pH showed a slight decrease with the concentration of the oil phase, registering values of 4.40, 4.72, and 4.73 for the emulsions containing 1, 3, and 5% pequi pulp oil (PPOE), respectively, for a period of 30 days according to the stability study carried out in this work (Figure 9). The pH of emulsions did not undergo significant variations with different concentrations of the oil phase, demonstrating the stability of the system. The greater system stability occurred when these values were maintained within a small pH variation, as proven in this study.

The correlation of the ζ analysis with the pH of the emulsions shows that, generally, the Zeta potential tends to become more negative with a decreasing pH [131,132,133]. This was used to justify the fact that the resulting ζ in this study was low in the pHs that were analyzed; that is, reaching negative load surface values of −25.20, −25.26, and −20.92 for the emulsions containing 1, 3, and 5% of pequi pulp oil, respectively. This indicated that the droplets present in the emulsions could have strong repulsive forces that prevented coalescence from occurring, maintaining system stability [134]. These negative charges could originate from the carboxylic acid groups present in the surfactant, which ionized, forming negatively charged droplets [135,136]. The ionization of these groups would depend on the pH of the system: the higher the parameter, the more negative the ζ [137].

The ζ measurement can be applied to assess the physical-chemical stability of emulsions and colloidal dispersions. Emulsions with values above +30 mV, as well as values below −30 mV, are considered stable. On the other hand, emulsions with values between −30 and +30 mV are unstable, with the value of 0 mV being the most unstable [47,138]. However, in addition to the electrostatic mechanisms, there may still be steric forces that prevent particle aggregation in the aqueous phase, regardless of the pH, as found in the work developed by de Barros et al. [139], who obtained stable systems with ζ values close to zero.

From the results shown in Table 3, the emulsions prepared in this study exhibited negative values; sufficient to stabilize oil-based emulsified systems of pequi pulp/polysorbate 80^®^/water. These negative values are a coating consequence of particles/emulsions with the polysorbate 80^®^ surfactant, having a negative surface charge density due to the presence of oxygen atoms in the molecules. The colloidal physical stability of these emulsions was due to the steric effect of surfactant (polysorbate 80^®^) at the particle/water interface [140]. 

A similar result was also observed by Marchiori et al. [141]. On the other hand, de Barros et al. [139] performed experiments using different pHs; at pH~5.5, they attributed the origin of the negative charge to the presence of carboxyl groups that had dissociated in the medium, resulting in a negatively charged surface.

Nonzero surface charge potentials will complicate instability processes, such as particle aggregation [142]. In the case of the present study, nonionic surfactants, such as the polysorbate 80 used in this study, favored the formation of a highly stable complex at the interface between the aqueous and oil phases. This complex forms a film responsible for particle repulsion by a steric hindrance mechanism [143]. The Zeta potential values ranging from −25 and −20 mV found in this study indicated the formation of stable physicochemical nanostructures due to the presence of a surfactant layer at the interface of each particle. Similar behavior was reported by Fiel et al. [144].

According to de Barros et al. [139], at any pH, the double thickness of the layer shrinks with the increasing ionic strength of the medium. Therefore, the surface potential decreases, and the stability of the colloidal particles is also diminished. In a pH 5.5 solution, the ionic strength was low; in a pH 1.2 solution, however, the ionic strength was higher. In our study, the emulsions containing 1, 3, and 5% pequi pulp oil and pHs ranging from 4.4 to 4.7 showed low ionic strength, favoring stability.

The values of the drops, hydrodynamic diameter, and the polydispersity index (PDI) of the emulsified formulations are displayed in Table 3. These attributes of the drops directly affect the physical stability of colloidal systems. The smaller the droplet size, the more stable the system will be. On the other hand, when the system presents agglomeration and/or coalescence of the droplets, they quickly increase in size, leading to instability [145,146].

The results obtained in the present work show that the formulations exhibited hydrodynamic diameters of 328.79, 140.10, and 95.93 nm, as well as polydispersity indexes of 0.35, 0.34, and 0.32 for the emulsions containing 1, 3, and 5% pequi pulp oil, respectively. Given these results, the tested formulations were characterized as nanoemulsions, presenting low values for the PDI, indicating low polydispersity for all systems. Similar behavior has been reported by Ombredane et al. [36] using egg lecithin as an emulsifier.

This behavior can be explained when adequate amounts of surfactants are used, leading to lower interfacial tensions during the homogenization process, facilitating the formation of smaller and more homogeneous nanodroplets [147,148].

According to Sanguansri and Augustin [149] and Gutiérrez et al. [150], nanoemulsions are considered true emulsions when there is one phase dispersed and one continuous, generally with a droplet diameter between 50 and 1000 nm.

The emulsion is transparent or translucent and has stability against sedimentation or other phenomena that lead to emulsified system instability.

It is important to highlight that the formulations analyzed in this study presented optical properties different from those reported in the literature when correlated with the mean particle diameter. The formulation, PPOE1, contained only 1% pequi pulp oil and presented, over the time of analysis, an average droplet diameter of 328.79 nm and a translucent appearance, as seen in Figure 9 below. This optical property was maintained over the entire 120-day period in the stability study. On the other hand, the formulations containing 3 and 5% pequi pulp oil (PPOE3 and PPOE5), respectively, presented the lowest mean diameter values of droplets (140.10 and 95.93 nm) and white coloration—a milky appearance such as that observed in Figure 3 (see above, in the section on macroscopic analysis as a function of time).

Finally, it is important to mention that all formulations prepared here remained physically stable over the 120 days of analysis, thus are considered viable for topical application.

### 3.8. Scanning Electron Microscopy (SEM)

**Figure 10:**
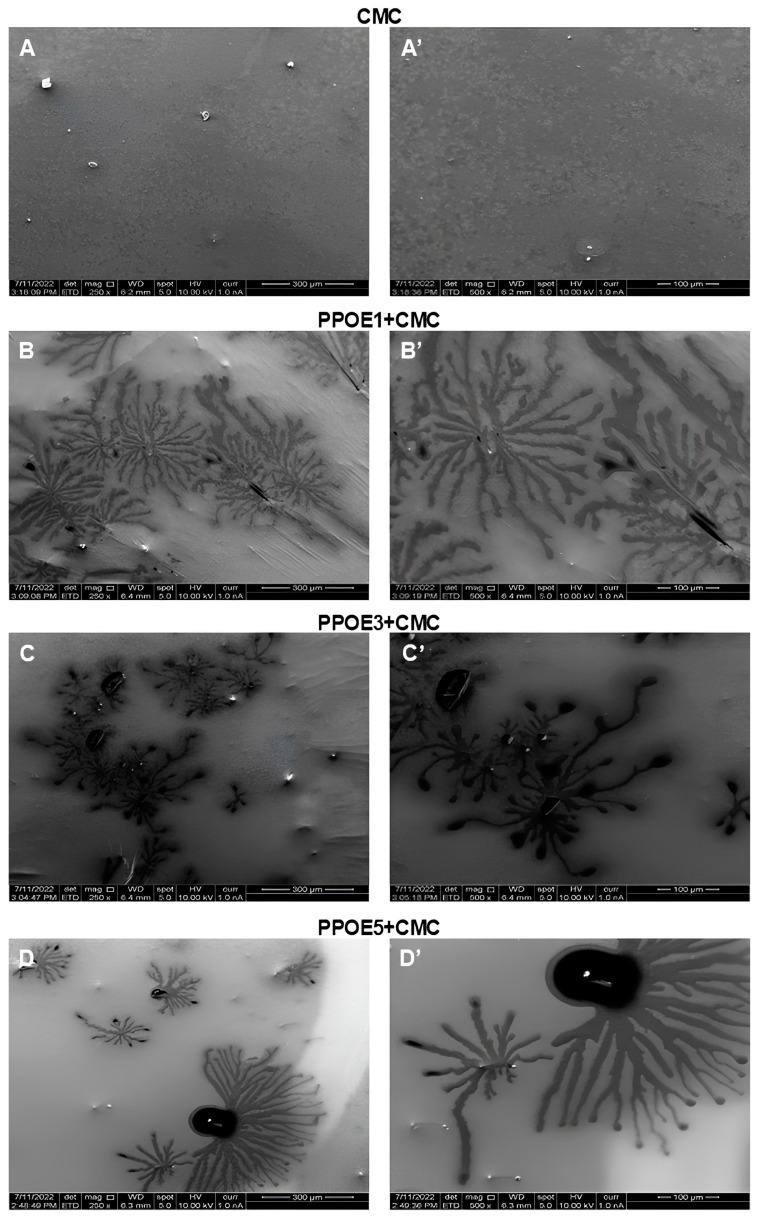
Micrographs of (**A**,**A’**) pure CMC and PPOE + CMC emulsions containing (**B**,**B’**) 1, (**C**,**C’**) 3, and (**D**,**D’**) 5% pequi pulp oil.

The microstructure and surface morphology of CMC membranes and PPOE + CMC emulsions containing 1, 3, and 5% pequi pulp oil were analyzed by scanning electron microscopy (SEM), and its micrographs are shown in Figure 10.

In the micrographs of the CMC membrane, a smooth surface is observed and continuously presents a variety of “spots” in light gray tones, with different formats and small, evenly distributed dimensions. These “stains” can be attributed to the presence of CMC lumps that were not completely solubilized during the preparation of the polymeric solution. This result corroborates the morphology analyzed by optical microscopy. The presence of some white dots can be seen, which is a possible characteristic of CMC crystallization or from the impurities of the polymeric solution or the drying process. In general, the morphology displayed by the CMC membrane is like that obtained by Akhtar et al. [151].

After the incorporation of CMC into the PPOE emulsions (PPOE + CMC) containing 1, 3 and 5% pequi pulp oil, a microstructural alteration could be observed according to the micrographs shown in Figure 10, in relation to the morphology of the pure CMC. In this case, a heterogeneous morphology and a certain roughness can be observed on the surface of the material, with the formation of microstructures with micelles shapes in varying sizes, undefined contours, and the presence of clusters in the form of filaments that vary with the oil concentration. The formation and presence of these micelles may be associated with the type of oil/water system and surfactant (polysorbate 80^®^) used, capable of promoting miscibility/compatibility between both emulsion components, promoting stability.

The PPOE1 + CMC sample exhibited smaller, uniformly sized micelles and a better distribution when compared to the other formulations that presented micelles with different sizes, irregular shapes, and non-uniform distribution along the continuous phase (PPOE3 + CMC and PPOE5 + CMC). These differences can be observed in the micrographs shown in Figure 10.

A greater number of filaments were observed in the samples containing 1 and 3% pequi pulp oil (PPOE1 + CMC and PPOE3 + CMC). Akhtar et al. [151] studied the incorporation of chickpea shell polysaccharide to carboxymethylcellulose (CMC) in levels from 0.25 to 1.00% and attributed the presence of these filaments to the retraction forces produced by the addition of chickpea hulls; they pointed out the dependency of these on the concentration used. A similar trend has been observed in our study when using pequi oil-based emulsions in CMC. In all micrographs of the formulations, the presence of white dots was also noted, possibly attributed to the crystal’s surfactant used in the preparation of the PPOE emulsions. 

Bertolino [152] reported in his study on membranes of chitosan/gelatin and pequi oil the presence of vesicles in varied sizes and without defined contours, as well as filaments with crystalline appearance. The increase in oil concentration reduced the vesicles’ size, increasing their uniformity. The presence of crystal-like filaments was also proportional to the pequi oil concentration. Therefore, it is suggested that the process used in the methodology above provides adequate compatibility and miscibility among membrane components.

## 4. Conclusions

Pequi pulp oil has antioxidant and anti-inflammatory compounds in its composition, essential for maintaining the integrity of the skin, making it potentially useful for applications in cosmetic formulations. All emulsions in this study showed physical/kinetic stability throughout four months. The type of surfactant, as well as the concentrations used, were crucial in stabilizing the emulsions (oil/surfactant/water), as well as the methodology used in the development of the samples. Carboxymethyl cellulose was efficient in the encapsulation process, providing essential characteristics to the emulsions, such as an increase in viscosity and better stability in the different systems studied. No instability was visible in this study over 8 months from when the formulations were prepared. The emulsions showed thixotropic and pseudo-plastic behavior, both desirable characteristics for pharmaceutical formulations for topical use. The pH of all emulsions was compatible with that of the skin. From these results, it is possible to suggest the potential use of pequi pulp oil encapsulated in CMC in the form of emulsions for use in the production chain of natural cosmetic and pharmaceutical products, given the desire for sustainable development. 

## Figures and Tables

**Table 1:** Fatty acids composition and other compounds present in pequi pulp oil (PPO).

Retention Time (min)	Compounds	Composition (%)	Molecular Formula
31.55	Oleic Acid	31.25	C_18_H_34_O_2_
32.90	Linoleic Acid	27.42	C_18_H_32_O_2_
28.03	Palmitic Acid	24.11	C_16_H_32_O_2_
31.88	Stearic Acid	9.65	C_18_H_36_O_2_
32.25	Malonic Acid	3.74	C_16_H_29_ClO_4_
29.00	Geraniol	2.85	C_20_H_34_O
45.65	γ-Tocopherol	0.75	C_28_H_48_O_2_
46.30	β-Sitosterol	0.22	C_29_H_50_O
TOTAL		100.00	

**Table 2:** pH of emulsions over a period of 120 days.

Sample	pH						
	Days						
	1	7	15	30	60	90	120
PPOE1	4.10	4.50	4.45	4.40	4.38	4.35	4.30
PPOE3	4.35	4.89	4.85	4.72	4.68	4.61	4.52
PPOE5	4.43	4.86	4.76	4.73	4.69	4.65	4.52
CMC	7.10	7.00	6.98	6.96	6.95	6.92	6.89
PPOE1 + CMC	6.78	6.75	6.69	6.65	6.63	6.63	6.58
PPOE3 + CMC	6.89	6.85	6.79	6.75	6.70	6.65	6.61
PPOE5 + CMC	6.85	6.80	6.76	6.71	6.67	6.55	6.50

**Table 3:** Zeta potential, droplet hydrodynamic diameter, and polydispersity index data for PPOE emulsions.

Sample	Zeta Potential (mV)	Droplet Hydrodynamic Diameter (nm)	Polydispersity Index
PPOE1	−25.20 ± 0.71	328.79 ± 3.48	0.35 ± 0.06
PPOE3	−25.26 ± 1.26	140.10 ± 4.95	0.34 ± 0.01
PPOE5	−20.92 ± 0.97	95.93 ± 1.85	0.32 ± 0.08

## Data Availability

The data are available upon request due to the volume of data and project limitations.
